# Big data integration shows Australian bush-fire frequency is increasing significantly

**DOI:** 10.1098/rsos.150241

**Published:** 2016-02-10

**Authors:** Ritaban Dutta, Aruneema Das, Jagannath Aryal

**Affiliations:** 1 CSIRO Data61, Hobart, Tasmania 7001, Australia; 2 School of Medicine, University of Tasmania, Hobart, Tasmania 7000, Australia; 3 School of Land and Food, University of Tasmania, Hobart, Tasmania 7001, Australia

**Keywords:** bush-fire frequency, ensemble machine learning, big data, climatic shift, decision science

## Abstract

Increasing Australian bush-fire frequencies over the last decade has indicated a major climatic change in coming future. Understanding such climatic change for Australian bush-fire is limited and there is an urgent need of scientific research, which is capable enough to contribute to Australian society. Frequency of bush-fire carries information on spatial, temporal and climatic aspects of bush-fire events and provides contextual information to model various climate data for accurately predicting future bush-fire hot spots. In this study, we develop an ensemble method based on a two-layered machine learning model to establish relationship between fire incidence and climatic data. In a 336 week data trial, we demonstrate that the model provides highly accurate bush-fire incidence hot-spot estimation (91% global accuracy) from the weekly climatic surfaces. Our analysis also indicates that Australian weekly bush-fire frequencies increased by 40% over the last 5 years, particularly during summer months, implicating a serious climatic shift.

## 1. Background and summary

Bush-fire is a complex climatic event that brings catastrophic consequences to the socio-economic environment of a country. Climate controls natural bush-fire events with very high cross-correlations [1–10]. However, it is essential to understand different correlations between a bush-fire incident and surrounding environmental conditions, before such an event can be predicted. The methodologies reported in the existing literature bring various biophysical measurements to estimate and parametrize the intensity, rate and severity of bush-fire and model the behaviour of bush-fire patterns [11–15]. Localized modelling of such complex phenomena is very expensive due to experimental costs, health and safety regulations for a simulated study. This type of area-specific or vegetation-specific modelling can only achieve limited generalization.

Main motivation of this research work was to bring heterogeneous Earth observation data sources together to establish bush-fire hot-spot estimation on a continental scale [16–25]. In this study, ensemble of deep learning (based on data-driven unsupervised deep belief neural network along with conventional supervised ensemble machine learning) was developed and successfully applied to predict bush-fire hot spots across the continent of Australia [26–30]. To the best of our knowledge, this is the first attempt to create an ensemble of deep learning and applied to solve a very topical and complex climatic problem of continental Australia.

In this study, machine learning approaches, including supervised and unsupervised learning techniques, were used to analytically establish the significant increment in Australian bush-fire frequency. Supervised learning is the machine learning task of generalizing a decision rule from a set of training instances. The physical interpretation of a set of classification results is as important as the algorithm itself. Hence, an unsupervised learning mechanism was developed to understand and formulate the supervised problem so that estimation and hot-spot estimation from the supervised stage would not have to be treated as a so-called ‘black box’ model.

In supervised learning, each instance consists of an input object (typically a vector) and a desired output value (also called the supervisory signal) that the input is mapped to. Supervised learning uses the training data to learn a function that can then be employed for inferring the output values of unseen input instances. This requires the learning algorithm to generalize from the training data to unseen situations in a ‘reasonable’ way. Based upon the natural grouping of data within the feature space, it is evident that supervised machine learning should be able to discriminate between the classes. Classification performance was rationalized on the basis of the knowledge learnt from this first unsupervised stage [17–20]. Combination of unsupervised and supervised machine learning has shown a great promise to have the capability of generalizing complex problems naturally [28–30].

Development of ensemble machine learning using deep learning is one of our key contributions in this research. We show that highly accurate hot-spot estimation of weekly bush-fire incidence is possible if estimated with the long-term weekly bush-fire frequencies as additional inputs to the data-driven machine learning-based modelling paradigm.

**Figure 1.**
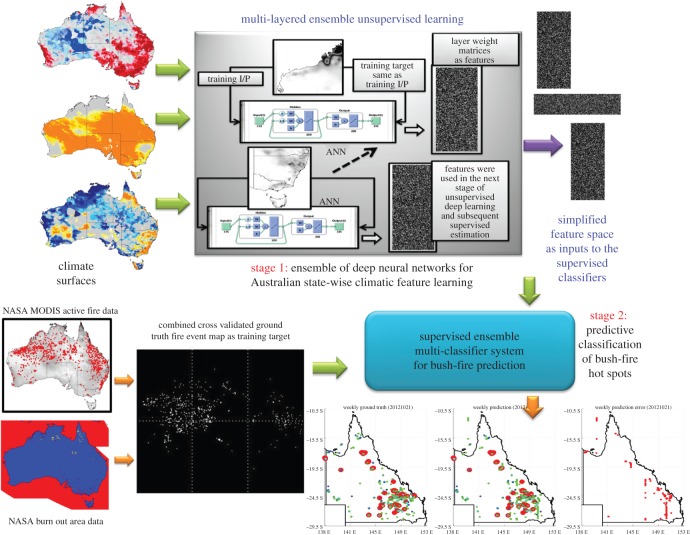
Ensemble deep learning mechanism for the weekly bush-fire frequency estimation and bush-fire weekly hot-spot estimation. This mechanism has two stages: the first one is the unsupervised deep learning phase and the second one is an ensemble supervised classification phase. In the unsupervised stage, multi-layered deep neural networks were employed to learn about the given data and generate simplified features without any prior information or training targets. In the ensemble classification stage, multiple supervised classifiers were used to learn the extracted features against the ground truth bush-fire host spot maps. Training inputs to the second stage are features (extracted in the first stage) from the climate maps along with the two estimated bush-fire frequencies (depicted in figure 2) based on the climatic zone boundaries and the administrative state boundaries on the Australian map. Outcome from this ensemble deep learning mechanism is a map with pixel (defined by latitude and longitude) based predicted bush-fire hot spots in multiple colours, according to the most probable category of the fire intensity or severity level (as defined in the electronic supplementary material, figures S2–S17). Maps and figures were generated using Matlab software packages. Copyright © CSIRO, Australia.

Further, bush-fire intensity distributions are used as additional contextual inputs to the two-layered ensemble deep learning mechanism (as depicted in figure 1). Bush-fire frequencies and intensities were estimated based on state boundaries and major climatic zone boundaries to conduct a comparative study considering the bush-fire management interests according to state boundaries and to depict the global picture from the climatic research interests.

It is evident that although new technologies are growing to manage the increasing number of unplanned bush-fires in Australia, but yet natural catastrophes cannot be tamed. From the recent history of Australian bush-fires, the most effective methodology to save life is believed to be early planned evacuation and early societal awareness and alerting. Early estimation of possible frequency of the future bush-fire incidence and hot-spot estimation of most probable bush-fire prone locations could provide great support to the land managers from the consequent damages [1–16]. In this research, we aim to develop a novel, data-driven, predictive and heterogeneous knowledge integration methodology to accurately predict bush-fire hot spots on a weekly temporal resolution and 250×250 m spatial resolution, which can provide great help to manage bush-fires in Australia [6–12]. Ultimately socio-economic impact of this research work could only be judged if a real system could be implemented as a decision support system, to accurately forecast most probable bush-fire hot spots on 250 m spatial and weekly temporal resolutions, for assisting Australian land managers to plan early evacuation to save lives and property.

## 2. Methods

In this study, NASA MODIS Active fire data product (based on satellite images from EOSDIS), Burned Area data product [31–33] and Australian Water Availability Project (AWAP) data [34] were integrated over a period of 336 weeks and experimented for accurate bush-fire incidence hot-spot estimation. In the initial unsupervised stage weekly AWAP climatic and environmental surfaces (15 variables namely solar radiation, maximum temperature, minimum temperature, daily rainfall, soil moisture, evaporation (soil and vegetation), total transpiration, soil evaporation, potential evaporation, local discharge (runoff and drainage), surface runoff, open water evaporation, deep drainage, daily sensible heat flux, and daily latent heat flux) along with weekly wind speed data [35,36] were captured together and integrated using a training paradigm of an unsupervised ensemble of deep neural networks (based on deep belief network constructed with several independent restricted Boltzmann machines), where no ground truth was presented to the networks [27,28,30]. Extracted weights from this unsupervised training stage were used to form an integrated and dimensionally reduced representation of the climatic data. These integrated weights were also used to tune the supervised learning phase, as described in the methods section of the electronic supplementary material.

### 2.1 Bush-fire incidence and intensity processing

**Figure 2.**
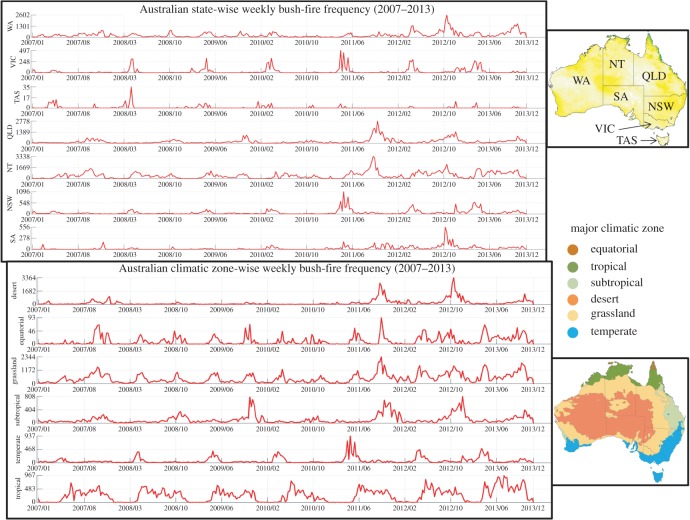
This figure represents the results about estimated Australian weekly bush-fire frequencies over the period 2007–2013 for each of the seven main administrative states, namely, Western Australia (WA), Victoria (VIC), Tasmania (TAS), Queensland (QLD), Northern Territory (NT), New South Wales (NSW) and South Australia (SA). Bush-fire frequencies were also estimated for the six major Australian climatic zones known as equatorial, tropical, subtropical, desert, grassland and temperate. Frequencies are estimated from NASA MODIS Active Fire data and NASA Burned Area data. Weekly frequency profiles of the states and climatic zones are integrated together with the AWAP climate data to train the ensemble deep learning mechanism. Bush-fire frequency information with weekly temporal resolution also provided the supervised classification stage an additional context to classify at very high hot-spot estimation accuracy with minimal false discovery rates. Weekly bush-fire frequency is defined as the total number of recorded fire events that occurred in a single week. On the *y*-axes are plotted the estimated actual fire frequencies. Quantification of the bush-fire frequency increment was calculated based on increased number of bush-fire hot spots on a weekly scale, which showed 40% increment over the last 7 years. Maps and figures were generated using Matlab software packages. Copyright © CSIRO, Australia.

**Figure 3.**
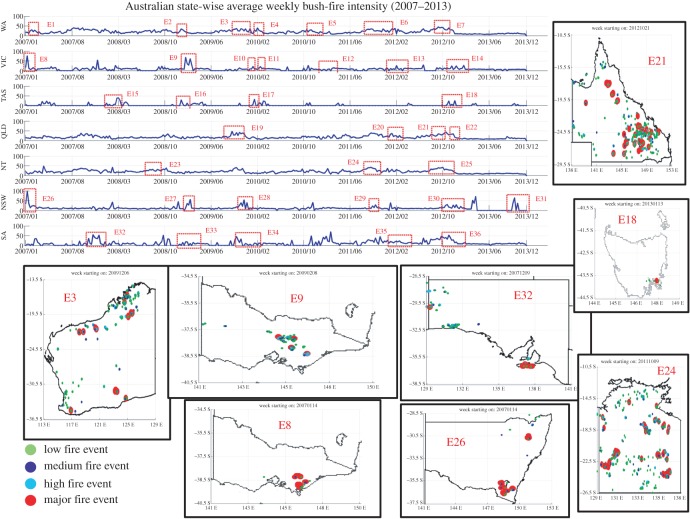
Representation of Australian state-wise average weekly bush-fire intensity derived from NASA MODIS Active Fire data and NASA Burned Area data. Weekly averaged and normalized fire intensities (in a range of [0–100]) are plotted for seven Australian states, WA, VIC, TAS, QLD, NT, NSW and SA. Historical verification and cross-validation of the derived bush-fire intensity data from NASA data products are performed. We have found 36 major fire events occurred during 2007–2013 (marked with red square and labelled with E1–E36) from various data sources [21–23], which are also present in the state-wise intensity profiles. To make the ground truth data used in this study more reliable, meaningful and understandable, we have visualized eight of the most severe bush-fire events in Australian history (E21, E18, E24, E3, E9, E32, E8 and E26) covering all of the seven states (short descriptions of other 28 major fire events are listed in the electronic supplementary material, tables S1 and S2). E21 represents September–October 2012 fire in QLD where several major fires occurred in a short span. E18 is one of the most severe bush-fires in recent Tasmanian history, where nearly 20 000 ha were burnt and 170 houses were destroyed in Forcett and Dunalley. E24 is NT bush-fire during September–October 2011 where 9000 km^2^ burned in Barkly and Victoria river region. E26 shows the major fire in Threadbo, NSW during January 2007. E3 represents many large fire events in WA during October–December 2009 (7 Mha burned in Kimberley). E32 shows the great fire of Kangaroo Island, SA in December 2007 (95 000 ha were burned). E8 (Great Gippsland fires in January 2007) and E9 (January–March 2009) are the two major bush-fire events in VIC. E9 represents many large and major fires, which includes ‘Black Saturday’, the most damaging bush-fire event in Australian history, killing 173 and injuring 414 people [15]. During E9 temperature in Melbourne city reached 46.4°C [15]. This figure depicts the correctness of our multiple source based integrated and pre-processed datasets used in this study. Maps and figures were generated using Matlab software packages. Copyright © CSIRO, Australia.

Because active fire products only provide information on fire location (fire line) at the moment of the image acquisition by the satellite and intensity (MODIS image intensity), these cannot provide accurate information about the actual extension and severity of the bush-fire detected. Burned area products provide information about bush-fire extent, but nothing about the bush-fire onset which is very important to estimate the climate and vegetation conditions under which the fire started to burn. Dry fuel is a major influence on fire incidence and spread that requires it to be included in any predictive modelling [1–15]. NASA Burned Area data product was used as a proxy for the effect of fuel level in the fire hot-spot area. NASA Active Fire and Burned Area data were blended together to cross-validate and refine the majority of actual fire events used as ground truth information [31–33]. Pixel value-based similarity comparison between weekly Active Fire product and Burned Area product images was used to find and establish the common areas (defined by a set of common latitude–longitude combinations at 250×250 m resolution) where bush-fire incidents were recorded. Based on available information about NASA's data observation quality, we filtered and used only bush-fire records with 50–100% confidence and averaged the close event location points to build the integrated dataset, to reduce the double counting issue in the estimated bush-fire frequencies. Weekly frequencies of fire incidence over 336 weeks (2007–2013) were estimated for seven Australian administrative states and also for the six major climatic zones [15,16] (as shown in figure 2). Bush-fire intensity is an estimate of the amount of energy released from the fuel by the fire as it burns. Active bush-fire brightness information from NASA Active Fire data blended with NASA Burned Area data were used to estimate weekly possible bush-fire intensity distributions for each of the seven Australian states (as shown in figure 2) over the same time period (as shown in figure 3). The bush-fire intensity was also derived cumulatively on a weekly temporal resolution. This was done primarily because we observed that in some cases weekly fire frequencies were low (number of fire events recorded per week) but recorded fire brightness was comparatively high, which was also validated by the evidence from the NASA Burned Area data from the following weeks. Another reason was to follow the convention of bush-fire measurement using its intensity. Estimation of weekly bush-fire intensities complemented the derived frequencies, as they are not linearly and proportionally correlated hence a data-driven approach is needed to establish the relationship in the context of bush-fire hot-spot estimations.

### 2.2 Ground truthing from the historical bush-fire records

We conducted extensive research in Australian bush-fire history to check the correctness of ground truth data based on 36 major bush-fire events as denoted by E1–E36 in figure 3 and in the electronic supplementary material, table S1. In Australia, there are two main vegetation types prone to fire. These are grasslands and forests. Therefore, a fire occurring in a grassy paddock is called a grass fire and a fire occurring in a forest is called a forest fire. Major fire events were collectively used to make our ‘Historical Ground Truth’ data more valuable and acceptable in the methodology.

Idea behind highlighting of these major fire events within our dataset was primarily to show that we are not just working on any data but cross-validated data according to the real historical facts. It was aimed that if the newly developed ensemble of deep learning mechanisms could predict the major events with higher generalization and repeatability, confidence in such a predictive system would be significant. From the historical event records, climatic conditions around the major Australian bush-fire were critical in our study, to capture as ground truth and model that information for future hot-spot estimations.

Electronic supplementary material, table S1 shows the integrated pieces of ground truth events, which we have used in our study for hot-spot estimation and cross-validation purposes. This is a unique step to make the data-driven deep learning-based approach more accurate and results are more easily interpretable. The electronic supplementary material, figures S2–S17 depict the detailed visualization of the most severe bush-fire events in Australian history (E21, E18, E24, E3, E9, E32, E8 and E26) covering all of the seven states [14–16].

### 2.3 Machine learning experiments

We used a gridded matrix of 704 rows and 817 columns for all input data sources. Six data segmentation regions were used for training and testing the network (electronic supplementary material, figure S1). The segments S1, S2, S4 and S5 have 335 rows and 300 columns. The segments S3 and S6 have 335 rows and 213 columns. The map-based image data (training inputs, testing inputs and training targets) were segmented into six smaller regions to maximize the learning speed while training and optimizing the computational memory usage.

Six dedicated deep belief networks (of size 100×100 hidden nodes using restricted boltzmann machines (RBMs)) for each of these segments (electronic supplementary material, S1–S6 in figure S1) were trained and weights were extracted individually. Reconstruction error from the RBMs was varied between 5 and 12%. Final estimated weight results (for all of the input climate surfaces) were constructed by combining the six segmented estimation outputs produced by the six individual networks. Size of the network was chosen based on an independent optimization process based on estimation performances, while main aim of this study was not to find the best size of a network, but to showcase a novel application of machine learning for a greater cause, e.g. bush-fire hot-spot prediction.

A typical pre-processed weekly dataset had 16 weight matrices (representing 15 climatic and environmental surfaces from AWAP and one weekly average wind speed as surface) extracted from the trained unsupervised deep belief networks as training and testing inputs for the supervised data experiments. The blended NASA products based ground truth bush-fire hot-spot locations (defined by latitude and longitude) were used to extract specific pixel values from the weight surfaces to create the input matrix (denoted by [A]). Randomly selected equally sized matrix of pixel values where no bush-fire was recorded was also added to the matrix [A] to have a balanced dataset.

The four bush-fire categories (C1, C2, C3 and C4 (as described in the Observations on Bush-Fire Frequency Increment section later)), were denoted by class labels 1, 2, 3 and 4, respectively, as training and testing targets for the supervised stage. These four labels were established by an incremental *k*-means clustering of the estimated weekly bush-fire frequencies and intensities, where objective function was defined by a 95% targeted information variance captured by the first component, after applying principal component analysis on the integrated frequency and intensity dataset.

Three different types of data experiments were conducted to test the generalization capability of proposed ensemble deep learning, namely: (i) the weekly datasets representing the whole Australian continent, (ii) the weekly datasets representing seven Australian states individually, and (iii) the weekly datasets representing six Australian major climatic zones. The training was done on the basis of randomly selected 50% of the total observations available in a dataset (covering 2007–2013 on a weekly temporal resolution) and the rest of the 50% observations were used for predictive testing. All together 14 predictive models (each of them had similar structures including two stages) were developed after several training and testing paradigms, to form an ensemble of deep predictors (six of them were for the six climatic zones, seven of them were for seven individual states and the last one was for the whole Australian continent). The testing outcomes from these models were called ‘hot-spot estimations’ as the corresponding testing inputs were unknown to the models during training phase. Our use of independent datasets for training and testing is the most widely used means of overcoming potential problems with overfitting. These strategies overcame the problem of the many imperfections of the MODIS fire incidence observations at the spatio-temporal scale of their collection.

Ten supervised classifiers including conventional supervised classifiers, namely, Binary Tree, Linear Discriminate Analyser, Naive Bayes, *k*-Nearest Neighbour (kNN), and ensemble classifiers called Bagging Tree, AdaBoosting Tree, Gentle Boosting Tree, Random Under Sampling Boosting Tree, Subspace Discriminant, and Subspace kNN were tried in stage 2 of the ensemble mechanism [26–30,37], to establish the best classifier for bush-fire hot-spot estimations comparatively. Methods were tuned using an independent optimization process based on individual methods’ estimation performances. The best estimation results are listed in the electronic supplementary material, table S2.

Effectiveness of the estimated feature sets from this unsupervised learning was judged based on the overall classification performance feedback from the next supervised stage (stage 2 in figure 1) using ensemble of multiple classifiers. Performance of these estimators was quantified by hot-spot estimation accuracy ((TP + TN)/(TP + FN + FP + TN) where TP = true positives, TN = true negatives, FP = false positives, FN = false negatives). The evaluation process also included sensitivity (TP/(TP + FN)); specificity (TN/(FP + TN); F1 scoring (2×TP/(2×TP+FP+FN)); and false discovery rate (FDR = FP/TP + FP) calculations in ascertaining the correctness of classification accuracies. More details and citations to the relevant references are provided in the Technical Validation section and added in the electronic supplementary material for greater clarity.

These models were trained to predict two outcomes from a combination of weekly feature inputs: (i) class label representing possibility of fire [0 = no bush-fire or 1 = bush-fire] and (ii) if bush-fire then possible category for the fire intensity from the category range C1–C4 (as described in the Observations on bush-fire frequency increment section). In the testing phase, we used all of these predictors to test each of the observations from the testing sets to find the most probable solution. The most probable predictive solution for a location was calculated based on the similar results predicted by the majority of the predictors for inputs from a given week. Novelty of this approach was in the final hot-spot estimation on the map, which was a derived outcome from an ensemble of predictors. In future scenarios, hot-spot estimation about probable bush-fire hot spots and fire frequency could be produced using these models and weekly climatic surfaces. The best model classifier, bootstrap aggregation (often abbreviated as bagging), uses each model in the ensemble vote with equal weight. In order to bring high level of model variance, bagging trains each model in the ensemble using a randomly drawn subset of the behavioural training set. Bagging works by training learners on resampled versions of the data. The minimal leaf sizes for bagged trees are set to 1. We found that conventional supervised ‘kNN classifier’ and ‘Bagging Tree’ were the two best classifiers with 91.76% and 94.53% global classification accuracies, respectively.

### 2.4 Observations on bush-fire frequency increment

Based on modified Köppen climate classification system [16] of the Australian continent's climatic zones, we have chosen six climatic zones. Estimated fire frequency profiles over 336 weeks revealed that tropical climate zone had almost periodic seasonal frequency distribution with significantly higher number of events, where maximum frequency was 967 events per week (results are shown in figure 2). It is well documented that different climatic zones and their characteristics have significant effects on bush-fire frequencies. Hence we took the initiative to mask different regions of the continent based on modified Köppen climate classification system to study the changing patterns of the fire events and fire frequencies over the last 7 years.

Based on the historical accounts on actual severities of the damage by the bush-fires, combined and correlated with all possible ranges of the derived ground truth fire intensities, we concluded that four main categories of bush-fire would be best to form a realistic classification problem and to develop the supervised predictive bush-fire classification and early alerting system.

The categorization of four bush-fire intensity thresholds was determined by applying simple *k*-means clustering on the whole integrated fire intensity dataset, where four clear clusters were formed [3–14,37]. The calculated centroids of these four clusters representing four naturally grouped fire intensities were used to define the four bush-fire categories. The four categories [37] were ‘Low fire event’ (C1; when MODIS bush-fire intensity is below 200), ‘Medium fire event’ (when MODIS intensity is between 200 and 500 (C2)), ‘High fire event’ (when MODIS intensity is between 500 and 900 (C3)) and ‘Major fire event’ (when MODIS intensity is above 900 (C4)).

The weekly ground truth data had the whole map of Australia with hot spots representing multiple categories of fires (as shown in the electronic supplementary material, figures S2–S17). This major climatic zone-wise frequency estimation along with the jurisdiction-wise estimation provided us a unique context to build the predictive model, which had bush-fire frequencies as additional contextual inputs.

The grassland zone had the highest number of fire events (average of 1067 per week), temperate (average of 428 per week) and subtropical (average of 378 per week) had similarly high-frequency distributions, whereas desert (average of 162 excluding stray events) and equatorial zones (average of 75 excluding stray events) had least fire frequency distribution (apart from some recent stray events where frequency reached a significantly higher value of 3364). Based on the correlation calculations over a long period of time, we find that weekly bush-fire frequency is heavily dependent on weekly average trends of soil moisture, solar irradiation, dry fuel and wind speed, and the specific geographical location within a major climatic zone [14–16,38,39]. Estimated average bush-fire event frequency over the whole continent of Australia was 3284 per week in 2007, in comparison with the frequency of 4595 events per week in 2013. This shows that weekly bush-fire frequencies for the Australian major climatic zones have increased by 40% since 2007. In particular, a major increase in bush-fire frequencies has been recorded from this data analysis since 2011/06 (figure 2) indicates a major climatic shift.

Outcome of that additional data pre-processing gave us unique contextual information to improve the ensemble deep learning based hot-spot estimation models. The final hot-spot estimations from the supervised ensemble model were fine-tuned using historical distribution of bush-fire frequencies, bush-fire intensities and context information related to the climatic demography of a probable bush-fire hot spot.

### 2.5 Code availability

Codes of this work are part of an ongoing project with industrial partners to take this work further, and hence are not freely available. However, we have integrated all weekly datasets into simple .mat file format to be used in Matlab environment. Descriptions of the data are included in the Data records section and public access has been provided through the figshare repository. Depending on interest and requirements some part of the code could be made available in Matlab scripting language for reading these data and conducting further experiments in Matlab.

## 3. Data records

Integrated public data have been made available as a single .zip file containing 336 .mat formatted files representing available 336 weeks between 2007 and 2013 (see Data citation section). A typical .zip file (of approx. 117MB) includes integrated weekly data from a particular year. One of these .zip files contains multiple .mat files, which could be easily accessed upon unzipping. Each of these files contains a total of 18 two-dimensional matrices of size 704×817 representing gridded data surfaces of 18 different climatic variables for the whole continent of Australia. These variables are Incoming Solar Irradiance (SolarMJ), Maximum Temperature (TempMax), Minimum Temperature (TempMin), Precipitation (FWPrec), Soil Moisture Upper Layer (WRel1), Soil Moisture Lower Layer (WRel2), Evaporation Soil+Vegetation (FEW), Transpiration (FWTra), Soil Evaporation (FWSoil), Potential Evaporation (FWPT), Local Discharge Runoff + Drainage (FWDis), Surface Runoff (FWRun), Deep Drainage (FWLch2), Sensible Heat Flux (PhiH), Latent Heat Flux (PhiE), NASA bush-fire ground truth hot spots (wf_gt_awap) and wind speed (wf_gt_wind); whereas total_wf_awap and total_wf_wind indicate total count of available bush-fire pixels and wind speed recordings. The reference coordinate system for the wind speed surfaces was # Longitude of lower left corner (xllcorner) 109.995°, Latitude of lower left corner (yllcorner) −45.005°, Cell size (Cell or grid size) 0.05. The reference coordinate system for the rest of the 17 surfaces was # Longitude of lower left corner (xllcorner) 112.925°, Latitude of lower left corner (yllcorner) −43.575°, Cell size (Cell or grid size) 0.05. All data files are formatted and loaded directly to be used in Matlab environment.

## 4. Technical validation

The weighted matrices of the trained neural networks were extracted as simplified representative features to be used in the next supervised classification (comprising of multiple supervised classifiers to form an ensemble) stage. State-wise weekly ground truth maps with bush-fire hot spots were used as training targets (as shown in figure 1). In this study, we used all the variables in the deep learning unsupervised phase to allow the neural networks to reduce data dimensions through natural selection. In this paper, we have shown that an ensemble of deep learning methodology is suitable for fire hot-spot estimation on a continental spatial scale and at a weekly temporal scale.

**Figure 4.**
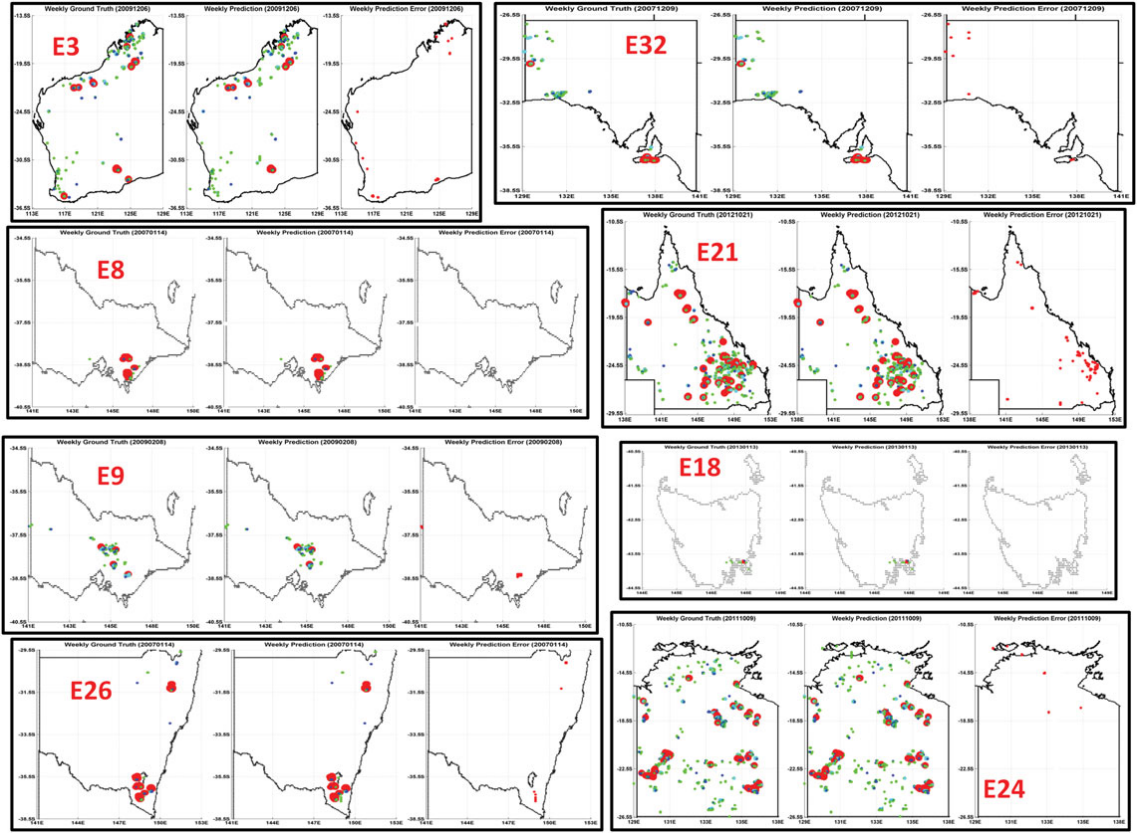
The ensemble deep learning approach based predicted maps with multiple categories of bush-fire hot spots for eight major fire events, as previously denoted with E21, E18, E24, E3, E9, E32, E8 and E26. Each of the images has three parts, ‘Weekly Ground Truth’ hot spots are on the left, ‘Weekly Hot-spot Estimation’ of the most probable hot spots in the middle and ‘Weekly Hot-spot Estimation Errors’ are on the right. The hot-spot estimation errors are marked with red dots to show the locations that are not successfullypredicted by the trained ensemble of deep learning mechanisms. Very high hot-spot estimation accuracies are clearly evident from all of these predicted maps with very low error rates in identifying weekly bush-fire hot spots. These results are visual representation of this novel predictive mechanism, whereas data from a total of 336 weeks (covering 2007–2013) have been studied and tested to calculate global accuracies, sensitivity, specificity and false discovery rate. Maps and figures were generated using Matlab software packages. Copyright © CSIRO, Australia.

This newly proposed methodology involving unsupervised ensemble deep learning mechanism together with a supervised ensemble of classifiers provided us with a high level of clarification. State-wise weekly fire incidence hot-spot estimations were tested against the ground truth scenarios as shown in figure 4. Total of 50% of the randomized weekly observations (including training inputs and training targets) were used to train the two-stage classifiers, where the rest of the 50% were used to test the accuracy performance. The predicted hot-spot patterns were closely similar to the observed patterns with very low error rates. The means for estimation accuracies for the 336 weeks (covering 2007–2013) were 91%, sensitivity 89%, specificity 94% and false discovery rate 6% [30,37,40].

Our aim was to create a predictive system that would allow calculation of the implications of climatic variation on bush-fire incidence within a weekly temporal scale. Such a system is potentially useful for both medium term (e.g. one month in the future) and long term (e.g. climate change models), but is not designed for daily hot-spot estimation [1–10,26,30]. However, the accurate hot-spot estimation of weekly fire incidence from weekly climatic data does not necessarily mean that human beings have little or no influence on fire incidence in Australia.

In temperate Australia, there is active discouragement of ignition, and attempts at suppression after fires are lit, during weather that could result in uncontrollable wildfire. There is less government interference in fire incidence in the tropics and the desert country. Prevention and suppression in temperate Australia may be having a spatially uniform effect on fire incidence, allowing climate to appear the sole influence. However, for the purpose of hot-spot estimation of the response of bush-fires to climate change, it may be safely assumed that the people of temperate Australia will not relax their vigilance in relation to summer fire, allowing our model to be used irrespective of the degree of anthropogenic influence, as long as that influence remains uniform within climatic regions.

A complete list of hot-spot estimation accuracies is provided in the electronic supplementary material, table S2. We conclude that ensemble deep learning mechanism could be a very effective and accurate way to predict weekly continental scale bush-fire hot spots, if modelled with weekly climatic surfaces in combination with the estimated historical bush-fire frequencies and intensities from the jurisdictional and climatic zones. Although learning and hot-spot estimation was done for the whole continent, we show a few examples of correct hot-spot estimations of major bush-fire incidents (as shown in figure 4 and electronic supplementary material, figures S2–S17) so as to make the hot-spot estimation more convincing and acceptable to the scientific community.

## Supplementary Material

ESM

## Data accessibility

1. Weekly integrated data for 2007: http://figshare.com/articles/Weekly_data_for_2007/1430502. 2. Weekly integrated data for 2008: http://figshare.com/articles/Weekly_data_for_2008/1430526. 3. Weekly integrated data for 2009: http://figshare.com/articles/Weekly_data_for_2009/1430524. 4. Weekly integrated data for 2010: http://figshare.com/articles/Weekly_data_for_2010/1430525. 5. Weekly integrated data for 2011: http://figshare.com/articles/Weekly_data_for_2011/1430576. 6. Weekly integrated data for 2012: http://figshare.com/articles/Weekly_data_for_2012/1430575. 7. Weekly integrated data for 2013: http://figshare.com/articles/Weekly_data_for_2007/1430577.
