# Supplementary material for: Big data integration shows Australian bush-fire frequency is increasing significantly
Source: R Soc Open Sci. 2016 Feb 10;3(2):150241. doi: 10.1098/rsos.150241 (PMC4785963; doi:10.1098/rsos.150241)

**Supplementary Information**

Big data integration shows Australian bush-fire frequency is increasing significantly

Ritaban Dutta^1*^, Aruneema Das^2^, Jagannath Aryal^3^

1 *CSIRO Data61, Hobart, Tasmania 7001, Australia*

*2 University of Tasmania, Hobart, Tasmania 7001, Australia*

3 *University of Tasmania, School of Land and Food, Hobart, Tasmania, Australia, Hobart, Tasmania 7001, Australia*

*** Corresponding Author, E-mail [*Ritaban.Dutta@csiro.au*](mailto:Ritaban.Dutta@csiro.au)

**Table S1:**

The complete list of the other 28 major bush-fire events (also denoted as E numbers on the Figure 4), which were also used to cross validate our weekly bush-fire ground truth hot spot data from the blended NASA products.

| **Bush-fire incidence Code from Figure 4** | **Time of the event** | **Australian State** | **Short Description including vegetation types and surface effected** |
| --- | --- | --- | --- |
| E1 | Feb 2007 | WA | >13000 hectares burned, south west of Perth fires |
| E2 | Dec 2008 – Mar 2009 | WA | >10000 hectares burned, Cape Le Grand National Park, Greater Perth, Mundijong, Dunsborough-Yallingup and Bridgetown fires |
| E4 | Mar 2010 | WA | >600 hectares burned, Coolup and Boronia Ridge fire |
| E5 | Nov 2010 – Feb 2011 | WA | >65000 hectares burned, lake Clifton, greater Perth, and Kimberley fires |
| E6 | Sept 2011 – Feb 2012 | WA | >95000 hectares burned, Margaret river, Nannup, and Northcliffe fires |
| E7 | Sept 2012 – Oct 2012 | WA | >5000 hectares burned, Madura station, Kimberly, Fitzroy, Toodyay and Parkerville and Two Peoples Bay fires, 1 casualty |
| E8 | Jan – Feb 2007 | VIC | >1.1 M hectares burned, Gippsland, Benalla and Ballarat fires |
| E10 | Dec 2009 | VIC | >7000 hectares burned, Port Nepean, Ballarat and Gippsland fires |
| E11 | Jan 2010 | VIC | >11000 hectares burned, Gippsland and Grampian fires |
| E12 | Feb 2011 | VIC | >8000 hectares burned, Gippsland fires |
| E13 | Jan 2012 | VIC | >230 hectares burned, Grampian fires |
| E14 | Jan 2013 – Mar 2013 | VIC | >90000 hectares burned, Kentbruck, Ballarat, and Aberfeldy fires |
| E15 | Jan 2008 | TAS | >1000 hectares burned, St Helens and Launceston |
| E16 | Jan 2009 | TAS | >5200 hectares burned, Epping fires |
| E17 | Jan 2010 | TAS | Swansea and St Helens fires |
| E19 | Sept 2009 –  Oct 2009 | QLD | Major fires in Gold coast, Rockhampton and Brisbane |
| E20 | Jan 2012 | QLD | Moreton island fires, burned for 4 weeks |
| E22 | Dec 2012 | QLD | 54 fires all over Queensland including Ipswich, Brisbane, Dalby, Cape York |
| E23 | Aug 2008 | NT | Savannah region fires |
| E25 | Aug 2012 –  Nov 2012 | NT | >100000 hectares burned, Newcastle, West MacDonnell Ranges, Curtin Springs Station, Napperby , Kings Canyon and Alice Springs fires |
| E27 | Feb 2009 | NSW | Tumut, Cabramurra, and Barmedman fires |
| E28 | Oct 2009 –  Feb 2010 | NSW | >40000 hectares burned, major fires Clarence Valley, Bathurst, Diamond Swamp, Gerogery, Tumbarumba, Wagga Wagga, Gundagai and Canberra suburb |
| E29 | Oct 2011 | NSW | Katoomba and Dudley fires |
| E30 | Nov 2012 –  Jan 2013 | NSW | >60000 hectares burned, Lake Macquarie, Hunter region, Murringo and Warrumbungle fires, 1000 sheep killed |
| E31 | Sept 2013 -Oct 2013 | NSW | Sydney and Blue Mountain fires |
| E33 | Jan 2009 | SA | Port Lincoln fires |
| E34 | Nov 2009 –  Jan 2010 | SA | Several fires across SA,NSW,VIC, and TAS, mainly Port Lincoln and Adelaide North fires |
| E35 | Jan 2012 | SA | >8000 hectares burned, Port Augusta fires |
| E36 | Nov 2012 –  Jan 2013 | SA | >5000 hectares burned, several 100 fires across the state, mainly Port Lincoln, Padthaway and Finniss fires |

**Table S2:**

The complete list of the prediction results for the 28 major bush-fire events (also denoted as E numbers on the Figure 4) using Bagging Tree ensemble system.

| **Bush-fire incidence Code from Figure 4** | **Time of the event** | **Australian State** | **Prediction Accuracy in terms of number of fire hot-spots** | **Specificity** | **Sensitivity** | **False Discovery Rate** |
| --- | --- | --- | --- | --- | --- | --- |
| E1 | Feb 2007 | WA | 95 | 89 | 95 | 6 |
| E2 | Dec 2008 – Mar 2009 | WA | 90 | 81 | 92 | 11 |
| E4 | Mar 2010 | WA | 92 | 90 | 90 | 7 |
| E5 | Nov 2010 – Feb 2011 | WA | 87 | 82 | 91 | 8 |
| E6 | Sept 2011 – Feb 2012 | WA | 85 | 95 | 93 | 10 |
| E7 | Sept 2012 – Oct 2012 | WA | 89 | 79 | 94 | 7 |
| E8 | Jan – Feb 2007 | VIC | 93 | 89 | 90 | 5 |
| E10 | Dec 2009 | VIC | 95 | 91 | 96 | 3 |
| E11 | Jan 2010 | VIC | 98 | 88 | 97 | 1 |
| E12 | Feb 2011 | VIC | 90 | 97 | 99 | 8 |
| E13 | Jan 2012 | VIC | 85 | 82 | 90 | 11 |
| E14 | Jan 2013 – Mar 2013 | VIC | 88 | 85 | 91 | 6 |
| E15 | Jan 2008 | TAS | 92 | 89 | 93 | 4 |
| E16 | Jan 2009 | TAS | 83 | 94 | 89 | 9 |
| E17 | Jan 2010 | TAS | 89 | 96 | 91 | 7 |
| E19 | Sept 2009 –  Oct 2009 | QLD | 91 | 93 | 92 | 6 |
| E20 | Jan 2012 | QLD | 95 | 83 | 95 | 3 |
| E22 | Dec 2012 | QLD | 90 | 87 | 97 | 9 |
| E23 | Aug 2008 | NT | 95 | 94 | 96 | 4 |
| E25 | Aug 2012 –  Nov 2012 | NT | 85 | 90 | 91 | 8 |
| E27 | Feb 2009 | NSW | 94 | 85 | 98 | 5 |
| E28 | Oct 2009 –  Feb 2010 | NSW | 97 | 86 | 97 | 2 |
| E29 | Oct 2011 | NSW | 98 | 94 | 94 | 1 |
| E30 | Nov 2012 –  Jan 2013 | NSW | 85 | 92 | 97 | 9 |
| E31 | Sept 2013 -Oct 2013 | NSW | 90 | 91 | 96 | 6 |
| E33 | Jan 2009 | SA | 98 | 86 | 92 | 1 |
| E34 | Nov 2009 –  Jan 2010 | SA | 94 | 80 | 88 | 3 |
| E35 | Jan 2012 | SA | 93 | 92 | 97 | 4 |
| E36 | Nov 2012 –  Jan 2013 | SA | 85 | 96 | 98 | 2 |

**Figure S1:**

The visualization of map format used for the climatic surfaces as input to the unsupervised deep learning phase. © CSIRO, Australia.


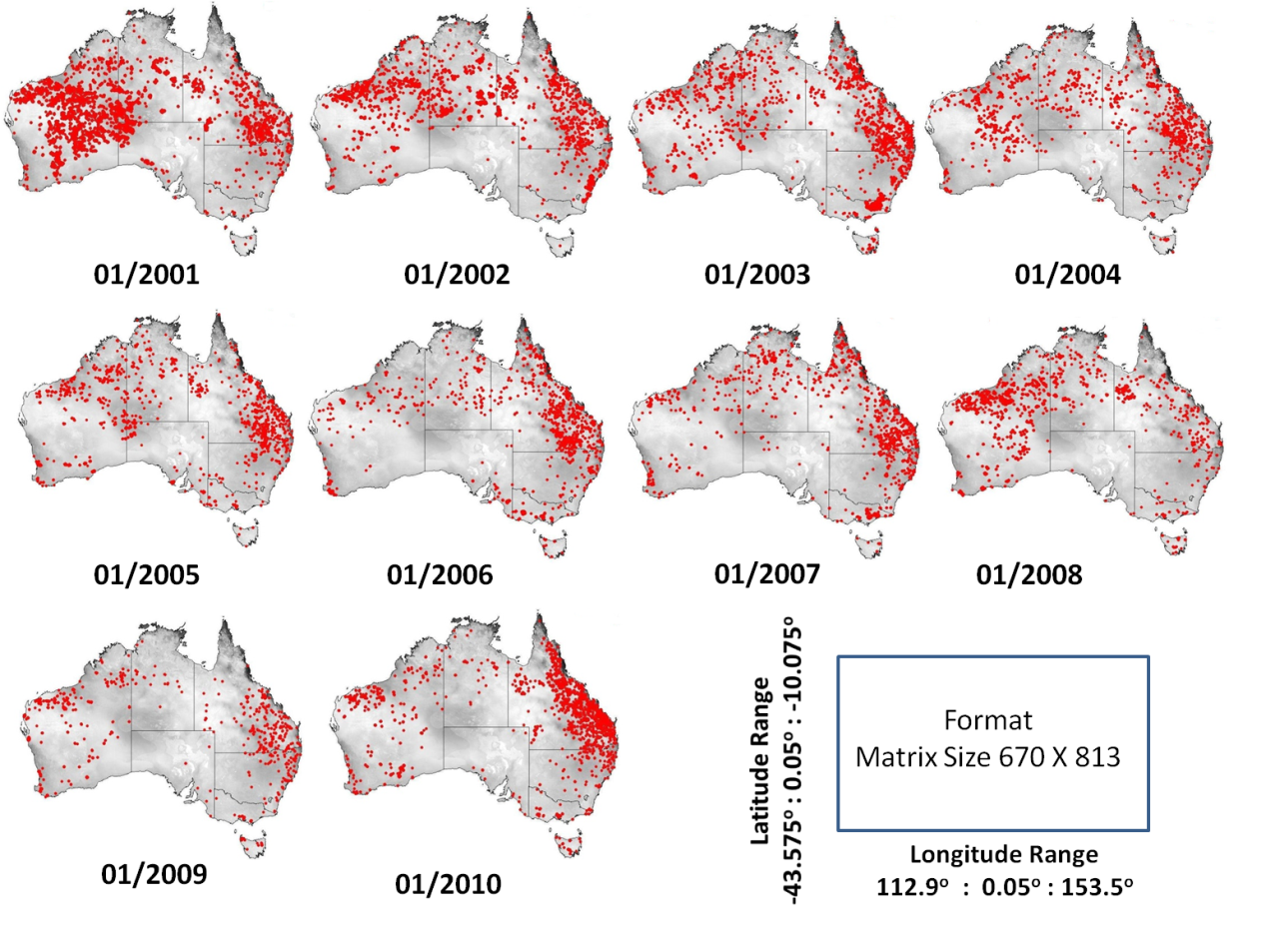


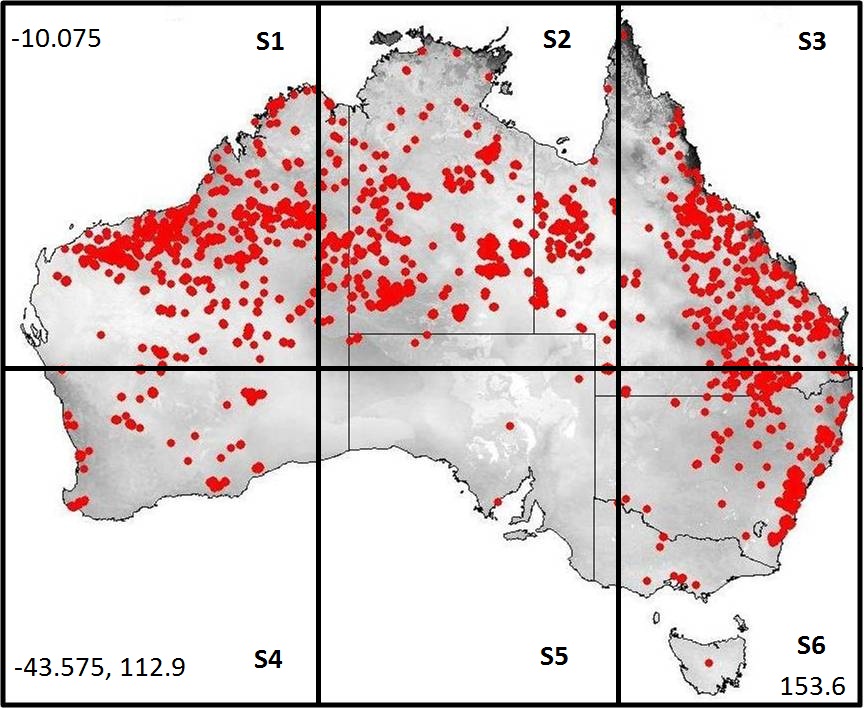


Figure S2:

The visualization of the severe E21 bush-fire event in QLD. Maps and figures were generated using MATLAB Software packages. © CSIRO, Australia.


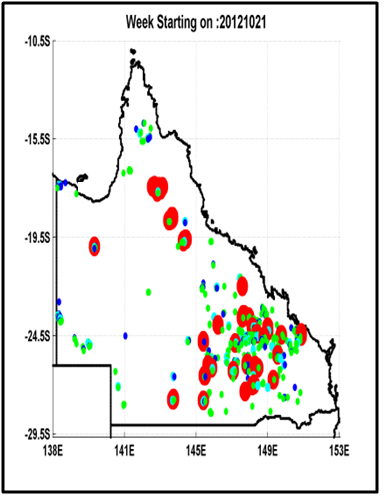


Figure S3:

The visualization of the severe E18 bush-fire event in TAS. Maps and figures were generated using MATLAB Software packages. © CSIRO, Australia.


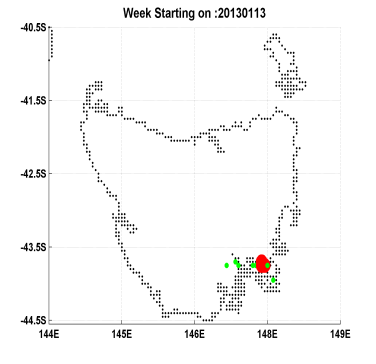


Figure S4:

The visualization of the severe E3 bush-fire event in WA. Maps and figures were generated using MATLAB Software packages. © CSIRO, Australia.


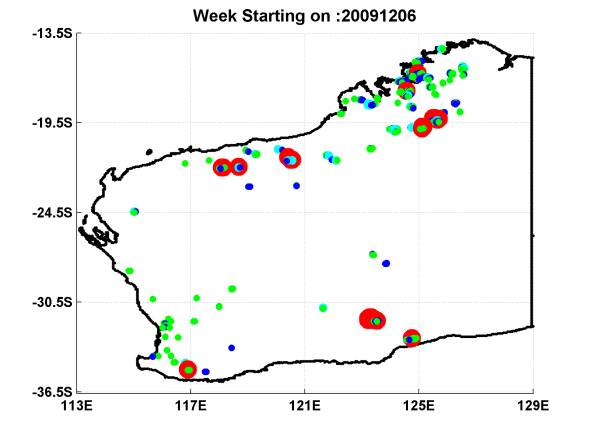


Figure S5:

The visualization of the severe E9 bush-fire event in VIC. Maps and figures were generated using MATLAB Software packages. © CSIRO, Australia.


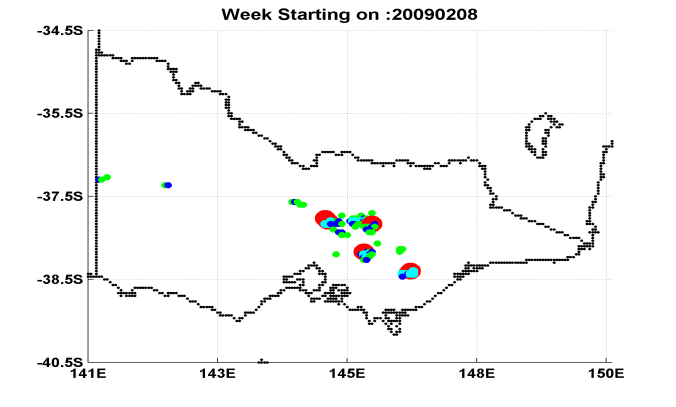


Figure S6:

The visualization of the severe E32 bush-fire event in SA. Maps and figures were generated using MATLAB Software packages. © CSIRO, Australia.


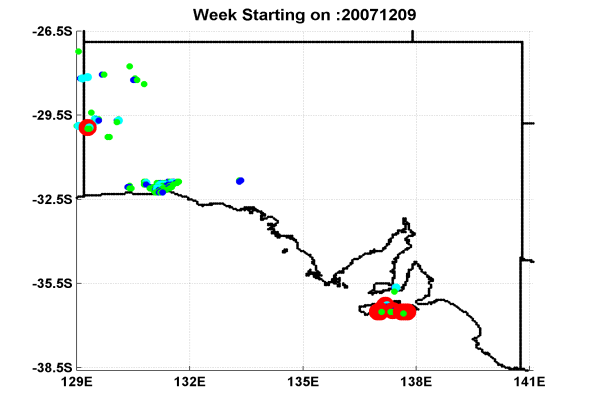


Figure S7:

The visualization of the severe E8 bush-fire event in VIC. Maps and figures were generated using MATLAB Software packages. © CSIRO, Australia.


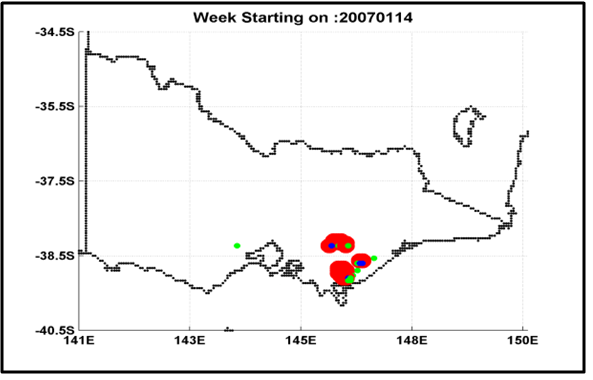


Figure S8:

The visualization of the severe E26 bush-fire event in NSW. Maps and figures were generated using MATLAB Software packages. © CSIRO, Australia.


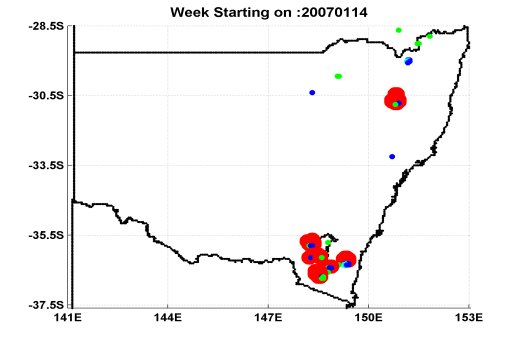


Figure S9:

The visualization of the severe E24 bush-fire event in NT. Maps and figures were generated using MATLAB Software packages. © CSIRO, Australia.


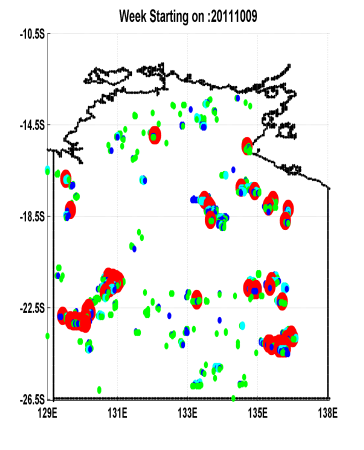


Figure S10:

The visualization of the prediction performance of E3 bush-fire event in WA. Maps and figures were generated using MATLAB Software packages. © CSIRO, Australia.


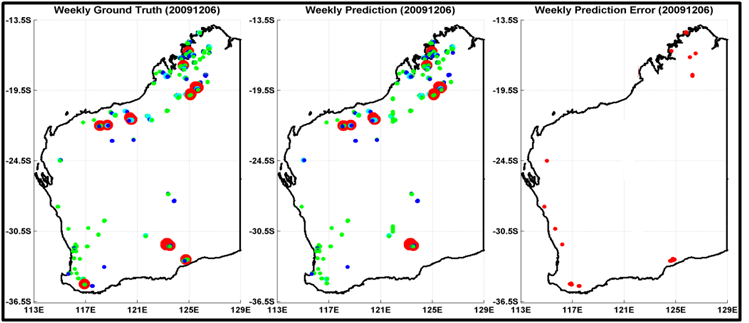


Figure S11:

The visualization of the prediction performance of E32 bush-fire event in SA. Maps and figures were generated using MATLAB Software packages. © CSIRO, Australia.


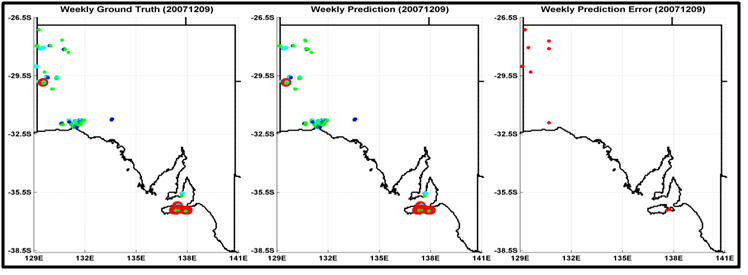


Figure S12:

The visualization of the prediction performance of E8 bush-fire event in VIC. Maps and figures were generated using MATLAB Software packages. © CSIRO, Australia.


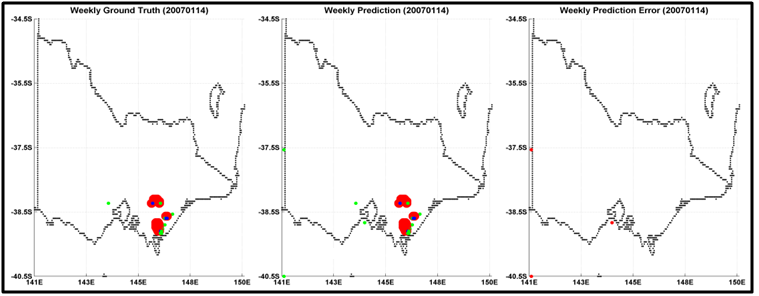


Figure S13:

The visualization of the prediction performance of E21 bush-fire event in QLD. Maps and figures were generated using MATLAB Software packages. © CSIRO, Australia.


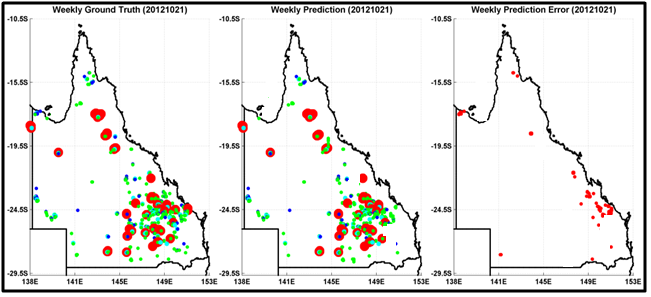


Figure S14:

The visualization of the prediction performance of E9 bush-fire event in VIC. Maps and figures were generated using MATLAB Software packages. © CSIRO, Australia.


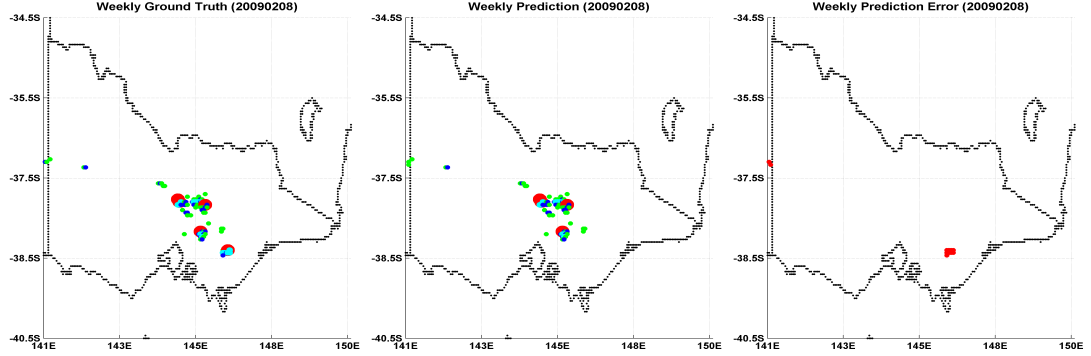


Figure S15:

The visualization of the prediction performance of E18 bush-fire event in TAS. Maps and figures were generated using MATLAB Software packages. © CSIRO, Australia.


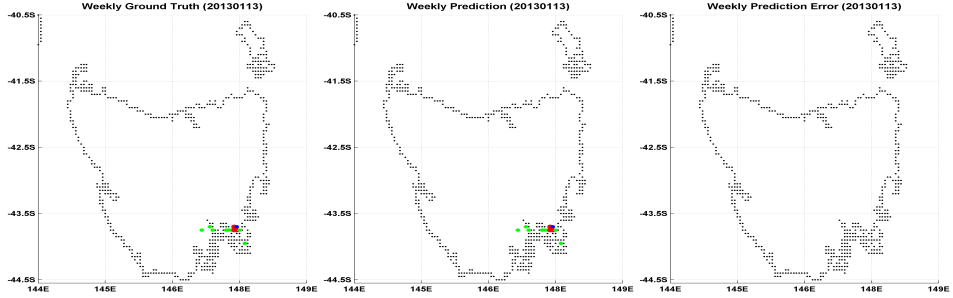


Figure S16:

The visualization of the prediction performance of E26 bush-fire event in NSW. Maps and figures were generated using MATLAB Software packages. © CSIRO, Australia.


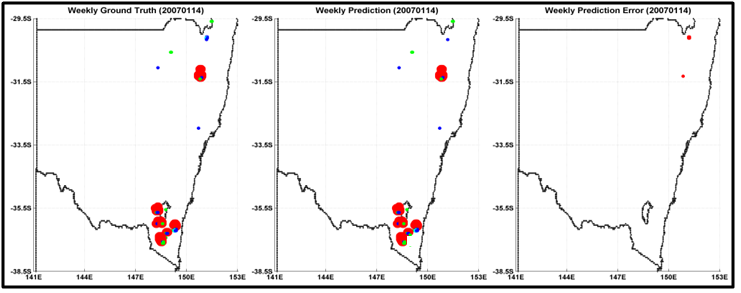


Figure S17:

The visualization of the prediction performance of E24 bush-fire event in NT. Maps and figures were generated using MATLAB Software packages. © CSIRO, Australia.


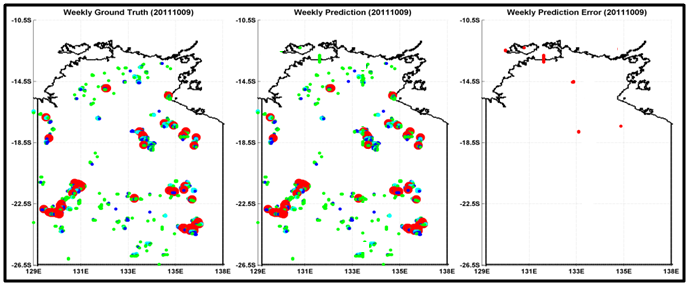

Supplement: ESM [file rsos150241supp1.doc]
